# Acknowledgment to Reviewers of *JDB* in 2020

**DOI:** 10.3390/jdb9010004

**Published:** 2021-01-29

**Authors:** JDB Editorial Office

**Affiliations:** MDPI AG, St. Alban-Anlage 66, 4052 Basel, Switzerland

Peer review is the driving force of journal development, and reviewers are gatekeepers who ensure that *JDB* maintains its standards for the high quality of its published papers. Thanks to the cooperation of our reviewers, in 2020, the median time to first decision was 15.1 days and the median time to publication was 38 days. The editors would like to express their sincere gratitude to the following reviewers for their precious time and dedication, regardless of whether the papers were finally published:
Ackley, BrianLevi, GiovanniAles, CveklMacNeil, Lesley T.Armant, OlivierMagarlamov, Timur Yu.Ashe, AlysonMaspero, CinziaAtukorallaya, DeviMccarthy, John J.Begemann, GerritMcClurg, UrszulaBialecka, MonikaMenzorov, Aleksei GBosveld, FlorisMitani, ShoheiBrites, PedroMorona, RuthBrunne, BiankaNavarro, NicolasCagnin, StefanoNavascues, Joaquin DeChelmonska-Soyta, AnnaOlson, EricChing, Tsui-TingPeronnet, FrederiqueCifuentes, DanielPettitt, JonathanCosta, VivianaPhilp, Nancy J.Crisponi, LauraPolesello, CédricDasgupta, SubhamPons, SebastianDash, SomaPruyne, DavidDawson, PaulQazi, Khaleda RahmanDe La Cova, ClaireRavanidis, StylianosDenham, MarkReddy, SakamuriDunnwald, MartineReynolds, ChristianDuprez, DelphineRichter, WitoDurand, Béatrice C.Robin, Nathaniel H.Dworkin, SebastianRogers, Melissa B.Everman, ElizabethSaint-Jeannet, Jean-pierreFish, Jennifer L.Sander, VeronikaGiantsis, IoannisSchellino, RobertaGogal, Robert M.Schlatt, StefanGrunow, BiankaSobierajska, KatarzynaHaenold, RonnySoto, Martha C.Henry, ClarissaSparagna, Genevieve C.Hiepen, ChristianSteiner, Florian A.Honore, StephaneStrathdee, DouglasHurley, James B.Tan, Tiong YangKanczler, Janos M.Tozer, SamuelKerosuo, LauraTse, William K.F.Khodanovich, MarinaVelasco, IvánKnudsen, Thomas B.Wake, David B.Kolesnikova, TatyanaWartiovaara, KirmoKrams, IndrikisWatt, KristinKumar, JustinWicky Collaud, ChantalKwan, KristenWoollard, AlisonLees, Simon J.Yetnikoff, LeoraLetra, AriadneZhao, Hui

